# Myosin VI maintains the actin-dependent organization of the tubulobulbar complexes required for endocytosis during mouse spermiogenesis^†^^‡^

**DOI:** 10.1093/biolre/ioz232

**Published:** 2020-01-04

**Authors:** Przemysław Zakrzewski, Anna Suwińska, Robert Lenartowski, Maria Jolanta Rędowicz, Folma Buss, Marta Lenartowska

**Affiliations:** 1 Department of Cellular and Molecular Biology, Faculty of Biological and Veterinary Sciences, Nicolaus Copernicus University in Torun, Torun, Poland; 2 Centre for Modern Interdisciplinary Technologies, Nicolaus Copernicus University in Torun, Torun, Poland; 3 Laboratory of Molecular Basis of Cell Motility, Nencki Institute of Experimental Biology, Polish Academy of Sciences, Warsaw, Poland; 4 Cambridge Institute for Medical Research, University of Cambridge, Cambridge, United Kingdom

**Keywords:** actin cytoskeleton, actin dynamics, fertility, myosin VI, spermiogenesis, tubulobulbar complexes

## Abstract

Myosin VI (MYO6) is an actin-based motor that has been implicated in a wide range of cellular processes, including endocytosis and the regulation of actin dynamics. MYO6 is crucial for actin/membrane remodeling during the final step of *Drosophila* spermatogenesis, and MYO6-deficient males are sterile. This protein also localizes to actin-rich structures involved in mouse spermiogenesis. Although loss of MYO6 in Snell’s waltzer knock-out (KO) mice causes several defects and shows reduced male fertility, no studies have been published to address the role of MYO6 in sperm development in mouse. Here we demonstrate that MYO6 and some of its binding partners are present at highly specialized actin-based structures, the apical tubulobulbar complexes (TBCs), which mediate endocytosis of the intercellular junctions at the Sertoli cell-spermatid interface, an essential process for sperm release. Using electron and light microscopy and biochemical approaches, we show that MYO6, GIPC1 and TOM1/L2 form a complex in testis and localize predominantly to an early endocytic APPL1-positive compartment of the TBCs that is distinct from EEA1-positive early endosomes. These proteins also associate with the TBC actin-free bulbular region. Finally, our studies using testis from Snell’s waltzer males show that loss of MYO6 causes disruption of the actin cytoskeleton and disorganization of the TBCs and leads to defects in the distribution of the MYO6-positive early APPL1-endosomes. Taken together, we report here for the first time that lack of MYO6 in mouse testis reduces male fertility and disrupts spatial organization of the TBC-related endocytic compartment during the late phase of spermiogenesis.

## Teaser

Myosin VI, a unique motor protein, is involved in the late phase of spermatid development by maintaining spatial integrity of testis-specific actin-rich structures—the apical tubulobulbar complexes.

## Graphical Abstract

**Figure:**
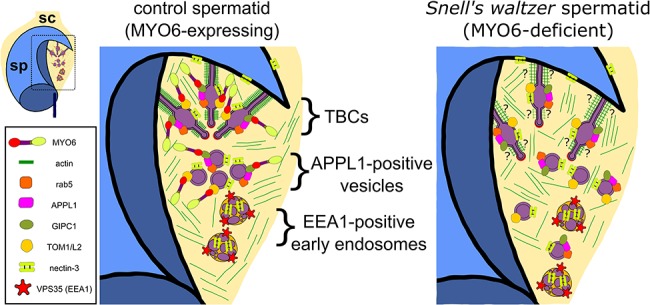

## Introduction

**Figure 1:**
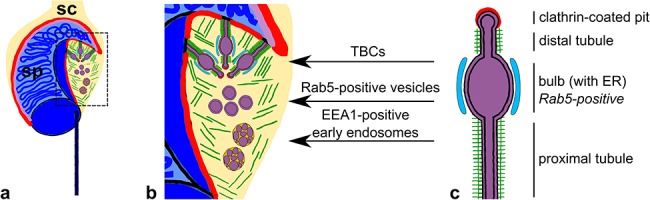
Schematic representation to highlight the organization of the TBCs during the late phase of mouse spermiogenesis. (a) Late spermatid surrounded by Sertoli cell cytoplasm. (b) Stages of the endocytic pathway linked to TBCs indicated with arrows. (c) TBC sub-compartments. *SC,* Sertoli cell; *SpT,* spermatid; *green lines—*actin filaments, *red line* in a/b—apical ES, *red line* in c—clathrin coat, *purple line* in b/c—acrosome, and *blue—*ER.

The formation of fully differentiated sperm is a result of dynamic morphological and biochemical changes that occur during spermiogenesis. In mammals, developing/maturing spermatids are surrounded by somatic Sertoli cell actin-based hoops that form a testis-specific adherens junction at the Sertoli-spermatid interface, known as the apical ectoplasmic specialization (ES) [1]. Functional organization of the apical ES and remodeling of its actin component is driven by the activity of different actin-binding/regulating proteins (ABPs), which enable the movement of spermatids in the seminiferous epithelium. At the late phase of spermiogenesis, the apical ES is replaced by highly specialized endocytic structure of membranes and actin filaments (AFs) called the apical TBC (Figure 1a and b). These unique complexes are formed at stage VII of the seminiferous epithelium cycle in mouse and have been suggested to play a role in the internalization and recycling of adhering junctions of the apical ES and thus are crucial for the sperm release during spermiation [2, 3]. The TBC consists of finger-like double-membraned invaginations of the Sertoli cell and the spermatid plasma membranes. The long neck of each invagination is stabilized and cuffed by an actin meshwork, followed by an actin-free region that extends into a bulb. This swollen bulb closely associates with the endoplasmic reticulum (ER) and terminates at the distal end in a clathrin-coated pit (Figure 1c). In addition to clathrin, two other marker proteins of the endocytic pathway have been localized to the TBCs—Rab5 [4] and its effector, the early endosomal antigen 1 (EEA1) [5]. Whereas Rab5 localizes to the bulbular region, EEA1 is present on large endocytic vesicles [6]. Thus, endocytosis of cell surface receptors for degradation or recycling might occur at the TBCs by the formation of Rab5-positive vesicles at the bulbs, which then fuse and mature into EEA1-positive early endosomes (Figure 1b). Indeed, it was recently suggested that junctional complexes are internalized at the apical TBCs to be recycled to newly formed intercellular junctions in other parts of the Sertoli cell [7]. Various ABPs involved in F-actin dynamics have been shown to drive the TBC formation/function, including actin cross-linker Arp2/3 complex and its activator cortactin, actin depolymerization factor cofilin, and actin regulators such as paxillin, vinculin, espin, Eps8/15 and N-WASP [8–10]. In addition, actin-based trafficking involving motor protein myosin VIIa has also been implicated in the apical ES/TBCs restructuring in mammals [11, 12]. Thus, the presence of the actin component and related proteins on these testis-specific structures during the late phase of spermiogenesis suggests that successful spermiation relies on the regulation of actin-based processes.

MYO6 is a unique motor protein, because unlike all the other myosins characterized so far, it moves towards the minus ends of AFs [13]. This myosin has been implicated in a wide range of cellular pathways including endocytosis, exocytosis, autophagy, maintenance of apical microvilli in polarized epithelial cells, and streocilia in inner ear hair cells as well as regulation of actin dynamics [14]. In these processes, MYO6 may function as a motor involved in intracellular transport or as an anchor essential in tethering membranes or protein complexes to the actin cytoskeleton. This unique ability results from MYO6 interactions with different cargo adaptors affecting its oligomerization [14]. For instance, during endocytosis, MYO6 may dimerize/oligomerize by interacting with GIPC1, regulating early endosomes positioning and cargo sorting [15]. On the other hand, MYO6 may act as a monomer while binding to TOM1/L2, thereby enabling tethering of endosomes to promote their fusion with autophagosomes [16]. A crucial role for MYO6 in *Drosophila* spermiogenesis, called spermatid individualization, was previously identified [17]. During this process, stable actin structures (cones) drive transformation of the syncytial spermatids into individual sperm by removing excess cytoplasm and membrane remodeling. MYO6 stabilizes a dense actin meshwork at the front of the cones as they move from the spermatid nuclei to the tails, which is required to complete spermiogenesis [17, 18]. The lack of MYO6 in *Drosophila* testis causes abnormal structure of the actin cones and loss of selected ABPs from the front of the cones and results in sterile male flies [19, 20]. These data suggest that MYO6 plays a structural role during *Drosophila* spermatid individualization. Our previous results also suggest a role for MYO6 in mouse spermiogenesis as this myosin is expressed in wild-type mice testes and localizes to actin-rich structures necessary for spermatid development/maturation, including the apical ES [21]. Moreover, it has been suggested that MYO6-deficient Snell’s waltzer (*sv/sv*) male mice show reduced fertility [22]. To further investigate the role of MYO6 in the late phase of spermiogenesis in mouse, we analyze here the impact of loss of MYO6 function on spatial organization of the TBC-related endocytic compartment using testis from the *sv/sv* and control mice. We identified defects in the actin cytoskeleton at the TBCs and the distribution of the APPL1-positive endosomes. Finally, we show significantly reduced litter size in Snell’s waltzer mice linked to male fertility.

## Materials and methods

### Animals

Three-months-old male Snell’s waltzer mice (C57BL/6 background) were used in this study. Each experiment was performed at least three times using a pair of control (heterozygous, *sv/+*) and mutant (*sv/sv*) males from one litter. All animal work was performed at the Nencki Institute of Experimental Biology (Warsaw, Poland) or at the University of Cambridge, Cambridge Institute for Medical Research (Cambridge, United Kingdom), where the mice were bred and housed under pathogen-free conditions. Animal housing and sacrifice procedures were performed in compliance with the European Communities Council directives adopted by the Polish Parliament (Act of 15 January 2015 on the use of animals in scientific investigations) and with the UK Animals (Scientific Procedures) Act 1986 and Laboratory Animal Science Association (LASA) Guidelines.

### Antibodies and reagents

The following primary antibodies for immunofluorescence (IF), immunogold localization (IG), immunoblotting (IB), and immunoprecipitation (IP) were used: rabbit polyclonal anti-MYO6 [23] (IF 1/50; IG 1/10; IP 5 μg); rabbit polyclonal anti-APPL1 (Proteintech 12639–1-AP; IF 1/100; IB 1/1000); mouse monoclonal anti-VPS35 (gift from Matthew Seaman, University of Cambridge, UK; IF 1/200); rabbit polyclonal anti-TOM1L2 (Abcam ab96320; IF 1/200; IG 1/50; IB 1/2000); rabbit polyclonal anti-GIPC1 (Proteintech 14822–1-AP; IF 1/100; IB 1/1000); mouse monoclonal anti-Arp3 (Sigma-Aldrich A5979; IF 1/200), mouse monoclonal anti-cortactin (Millipore 05–180; IF 1/100); rabbit polyclonal anti-PVRL3/nectin-3 (Proteintech 11213–1-AP; IF 1/100); and rabbit polyclonal anti-actin (Sigma-Aldrich A2066; IB 1/2000). The specificity of the primary antibodies against MYO6 and its partners was validated by western blotting of protein lysates from mouse testes. The secondary antibodies used for IF were goat anti-mouse/rabbit IgG (H + L) and Alexa Fluor 488/568 (Invitrogen). Protein A gold conjugates (Department of Cell Biology, University of Utrecht, Netherlands) were used to detect primary antibodies in IG approach. The secondary anti-mouse/rabbit antibodies used for IB were HRP-conjugated (Sigma). F-actin was visualized using Alexa Fluor 488 Phalloidin (Invitrogen). Nuclei were counterstained with Hoechst 33342 (Thermo Scientific). Normal rabbit IgG (Sigma-Aldrich) was used as a negative control during IF and IG experiments.

### Preparation of epithelial fragments

Dissected testes from *sv/+* and *sv/sv* males were decapsulated and minced in 4% (v/v) formaldehyde in 1 × PBS (pH 7.4) and left overnight at 4 °C. Next, seminiferous tubule segments were aspirated gently through 18-gauge and 21-gauge syringe needles [24]. Larger fragments of tissue were allowed to settle to the bottom of the tube, before the supernatant was removed and centrifuged (1 min at 4000 × *g*). The pellet was resuspended in PBS, and the cell suspension was added onto poly-L-lysine-coated coverglasses. After 10 min, the slides were plunged into ice-cold acetone and air-dried. Using this technique, we were able to obtain stage VII spermatids with the cytoplasm of Sertoli cell attached at the concave area of spermatid head where TBCs are formed.

### Immunofluorescence studies

For immunolocalization studies, the fixed samples were blocked with 1% (m/v) BSA in 1 × PBS, pH 7.4 supplemented with 0.1% (v/v) Triton X-100, and then incubated overnight at 4 °C with primary antibodies in 1% BSA/PBS followed by secondary antibodies conjugated with different fluorochromes, and DNA was stained with Hoescht. For negative controls, the samples were incubated with normal rabbit IgG instead of the primary antibodies. Epi-fluorescence images were captured on Zeiss Axio Imager.Z2 upright microscope, Airyscan high-resolution images on Zeiss LSM 880 laser scanning microscope, and confocal images on Leica TCS SP8 laser scanning microscope. Acquired images were processed with Zeiss ZEN 2.6 (blue edition), Leica LAS AF, Fiji [25], and Adobe Photoshop CS6 software. All representative images reported here were from at least three pairs of *sv/+* and *sv/sv* male littermates in three independent experiments, *n* = 3 with 2 testes used from each male.

### Immunogold electron microscopy

For post-embedding immunogold localization, dissected *sv/+* testes from *n* = 3 mice were fixed with a mixture of 4% (v/v) formaldehyde and 0.25% (v/v) glutaraldehyde in 1 × PBS (pH 7.4) for 2 h at room temperature. To facilitate penetration of the fixative, the tunicae albugineae of the testes were punctured several times with a syringe needle. Pre-fixed testes were then cut into small pieces (about 2 mm^3^) and fixed in the same fixative overnight at 4 °C. Next, the tissue fragments were dehydrated through a series of increasing ethanol concentrations and embedded in LR Gold resin (Sigma-Aldrich) according to the standard protocol. Samples were then cut with a diamond knife into ultrathin sections and collected onto nickel grids. The sections were blocked with 1% (m/v) BSA in PBS (pH 7.4) for 5 min at room temperature. Next, the samples were incubated with primary antibodies in 0.1% (m/v) BSA in PBS overnight at 4 °C, followed by 60 min incubation with a 10 nm gold-conjugated protein A diluted 1:50 in 0.1% BSA in PBS at room temperature. For double immunogold labeling, sections were incubated with the first primary antibody and then with the 10 nm gold-conjugated protein A according to the same protocol. Next, sections were post-fixed with 1% (v/v) glutaraldehyde in PBS for 5 min at room temperature and incubated again with the second primary antibody, and then with the 15 nm gold-conjugated protein A (at the same time and temperatures as for the first antibody). For negative controls, the sections were incubated with normal rabbit IgG instead of the primary antibodies. Finally, the sections were post-stained with 2.5% (m/v) uranyl acetate and 0.4% (m/v) lead citrate aqueous solutions, and examined and imaged on a FEI Tecnai G2 Spirit BioTwin transmission electron microscope equipped with a Gatan CCD Camera. Acquired images were processed with Adobe Photoshop CS6. All representative images reported here were from experiments repeated at least three times.

### Immunoblotting

Testes dissected from *sv/+* and *sv/sv* males (*n* = 3 for each phenotype) were homogenized with a Dounce tissue grinder in protein extraction buffer (50 mM Tris-HCl pH 7.5, 0.5% Triton X-100, 150 mM NaCl, 5% glycerol) supplemented with 1 × cOmplete Protease Inhibitor Cocktail (Roche). The homogenates were centrifuged twice at 15,000 × *g* for 10 min at 4 °C, before determining protein concentrations of the supernatants using a Bio-Rad DC Protein Assay according to the manufacturer’s instructions. Equal amounts of protein extracts were separated by electrophoresis on 12% SDS–PAGE gels and transferred to Amersham PVDF Hybond-P membranes (GE Healthcare), which were incubated with primary antibodies overnight at 4 °C, washed, and probed for 1 h with the corresponding anti-rabbit IgG or anti-mouse IgG/IgM secondary antibodies conjugated to horseradish peroxidase. Signals were detected using the Amersham ECL Advance Western Blotting Detection Kit according to the manufacturer’s guidelines (GE Healthcare). All immunoblotting experiments were repeated three times.

### Co-immunoprecipitation

Testes dissected from *sv/+* males were homogenized with a Dounce tissue grinder in ice-cold lysis buffer (50 mM Tris-HCl pH 7.4, 150 mM NaCl, 5 mM EDTA, 5 mM MgCl_2_, 1% NP-40, 5 mM ATP) supplemented with 1 × cOmplete Protease Inhibitor Cocktail (Roche) and centrifuged at 15,000 × *g* for 10 min at 4 °C. The lysates were pre-cleared with Protein A-Sepharose CL-4B (GE Healthcare) for 1 h at 4 °C and spun briefly, and then supernatants were transferred to fresh tubes. Next, samples were incubated with 5 μg of anti-MYO6 antibody for 1 h at 4 °C with end-over-end mixing, before incubation with Protein A-Sepharose for 1 h at 4 °C followed by four washes with ice-cold lysis buffer. Co-immunoprecipitated proteins were eluted from the beads using 4 × SDS sample buffer and analyzed by SDS-PAGE followed by western blotting. The primary antibodies were detected using Clean-Blot IP Detection Reagent (Thermo Scientific). Co-immunoprecipitation experiments were repeated three times.

### Statistical analysis

Each experiment was conducted at least three times on pairs of *sv/+*and *sv/sv* littermates. The obtained results were presented as the mean ± S.E.M. The statistical significance in each experiment was analyzed using an unpaired two-tailed Student’s *t*-test. The data were considered significant when *P* < 0.05. All data analyses were performed using GraphPad Prism 6 for Windows.

## Results

### Male mice lacking MYO6 expression are sub-fertile

**Figure 2:**
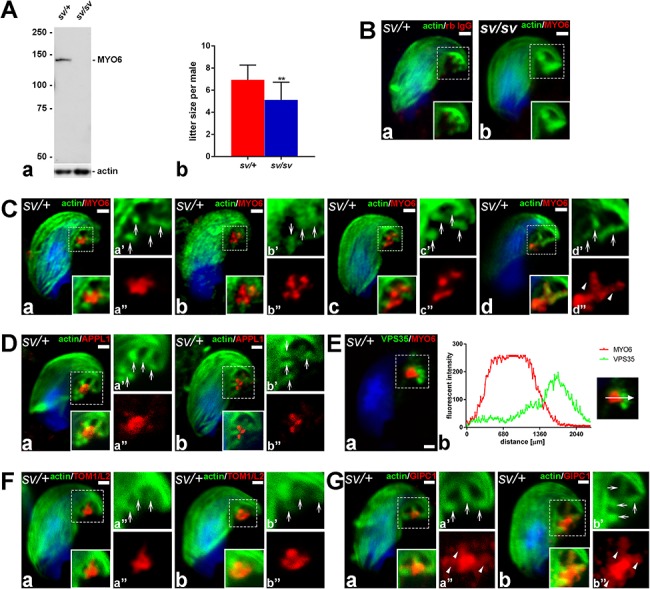
MYO6 localizes together with TOM1/L2 and GIPC1 to APPL1-positive vesicles at TBCs. (A) Immunoblotting of cell extracts from *sv/+* and *sv/sv* testes with anti-MYO6 and anti-actin antibodies (a). Graph showing the quantification of the mean litter size per *sv/+* and *sv/sv* male mouse (b). Both *sv/+* (*n* = 14) and *sv/sv* (*n* = 16) males were bred with *sv/+* females. ^**^P ≤ 0.01. (B) Stage VII *sv/+* spermatid treated with normal rabbit IgG (rb IgG, *red*) instead of the primary antibodies (a) and stage VII *sv/sv* spermatid incubated with anti-MYO6 antibody (*red*) (b). F-actin was visualized with fluorescently labeled phalloidin (*green*). (C) Confocal images of *sv/+*stage VII spermatids immunostained for MYO6 (*red*) and F-actin (*green*). The boxed area shown in (a) is enlarged and shown in single color in a’ and a”. (D) Confocal microscope images of *sv/+*stage VII spermatids immunostained for APPL1 (*red*) and F-actin (*green*). (E) Confocal microscope image of stage VII *sv/+* spermatid immunostained for MYO6 (*red*) and VPS35 (*green*) (a) and linear fluorescence intensity values across the TBC region showing the relation between MYO6- and VPS35-positive sub-compartments (b). (F) Confocal microscope images of stage VII *sv/+* spermatids immunostained for TOM1/L2 (*red*) and F-actin (*green*). (G) Confocal microscope images of stage VII *sv/+* maturing spermatids immunostained for GIPC1 (*red*) and F-actin (*green*). *Arrows* highlight the position of the TBCs, *arrowheads* indicate co-localization between MYO6 or GIPC1 with actin-positive regions of the TBCs, and *dotted squares* indicate areas magnified in images marked with prime and double prime. *All bars* 1 μm.

To determine whether loss of MYO6 expression in mouse testes (Figure 2A.a) affects reproductive functions, we calculated the average litter size of *sv/+* or *sv/sv* male mice crossed with *sv/+* females (Figure 2A.b). For statistical analysis, we used data gathered from litters generated during 2 years of mice breeding at stable and pathogen-free environmental conditions. Our data shows that the average litter size for *sv/+* male was 6.94 ± 1.33 (*n* = 14), whereas for *sv/sv* males was 5.12 ± 1.60 (*n* = 16). Thus fertility of *sv/sv* male mice was reduced by about 26% (*P* = 0.0022).

### MYO6 together with its binding partners, TOM1/L2 and GIPC1, localizes to APPL1-postitive vesicles at TBCs

The highly specialized tubulobulbar endocytic compartment mediates the uptake of cell-cell junctions in double membrane vesicles into Sertoli cell to allow sperm release in mammals. Since MYO6 is involved in endocytosis in various cell types and tissues [14, 26] and depletion of this myosin leads to reduced male fertility in mice (Figure 2A.b), we first assessed MYO6 localization during the late phase of mouse spermiogenesis. Using immunocytochemistry and high-resolution fluorescence microscopy, we detected endogenous MYO6 in the area adjoining the concave part of the spermatid head (stage VII tubule) in heterozygote *sv/+* control mice (Figure 2C.a–d). The strongest MYO6 signal was found at the center of the TBCs cluster in the Sertoli cell cytoplasm (Figure 2C.a”), which can be identified by the linear F-actin cuffs (Figure 2C.a’, *arrows*). In some sample preparations, high-resolution imaging enabled us to resolve separate MYO6-positive spots (Figure 2C.b” and c”), which are likely to represent distinct vesicles in close proximity of the TBCs (Figure 2C.b’ and c’; *arrows*). In addition, we observed labeling for MYO6 along the actin rich regions surrounding the extended necks (Figure 2C.d”, *arrowheads*). No specific signal at the TBC compartment was observed in the *sv/+* spermatids incubated with normal rabbit IgG instead of the primary antibody (Figure 2B.a) or in the *sv/sv* spermatids labeled with anti-MYO6 antibody (Figure 2B.b).

To further analyze the nature of the MYO6-containing vesicles, we labeled spermatids with an antibody for the endosomal marker APPL1, which is present on a specific class of early signaling endosomes that do not contain EEA1. APPL1 is an effector of Rab5, which is also present at TBC bulbs. APPL1 recruits MYO6 to early endosomes by binding directly to the MYO6 adaptor protein GIPC1 [6, 27]. Interestingly, the localization of APPL1 was very similar to the distribution of MYO6; both proteins were concentrated at the focus point of the TBCs (compare Figure 2C.a–a”and D.a–a” ) and sometimes appeared in a vesicular pattern (compare Figure 2C.b–b” and D.b–b”). To determine more precisely the nature of the MYO6-positive endocytic compartment that is associated with the TBCs, we next labeled maturing spermatids with an antibody to VPS35 (vacuolar protein sorting-associated protein 35), a subunit of the retromer complex involved in retrograde transport of proteins from endosomes [28]. VPS35 is a marker protein found on early sorting endosomes that also contain EEA1, which has been previously shown to be localized near the ends of TBCs [6]. Double labeling experiments for VPS35 and MYO6 showed very little overlap (Figure 2E.a), indicating that the MYO6 and APPL1-positive vesicles form a different endosomal sub-compartment that is distinct from EEA1-positive endosomes. These results are quantified in Figure 2E.b by the graph depicting linear pixel values across the line marked with *white arrow*. Taken together, our results suggest that MYO6 is involved in an early stage of the endocytic process at the TBCs and is present on APPL1-positive early endosomes that are different from EEA1-containing early sorting endosomes.

**Figure 3:**
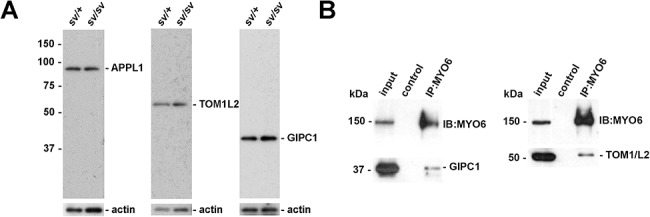
MYO6 forms a complex with TOM1/L2 and GIPC1 in mouse testes. (A) Immunoblotting of tissue extracts from *sv/+* and *sv/sv* testes with anti-APPL1, anti-TOM1/L2, anti-GIPC1, and anti-actin antibodies. Actin is shown as a loading control. No changes in protein expression were observed in *sv/sv* spermatids in comparison to control cells. (B) Co-immunoprecipitation of MYO6, GIPC1, and TOM1/L2 from *sv/+* testes lysates. MYO6 was immunoprecipitated with a rabbit polyclonal antibody. Lysates of *sv/sv* testes were used in the control lane. The immunoprecipitates were analyzed by western blotting with antibodies to GIPC1 and TOM1/L2*.*

We next determined which of the known adaptor proteins may interact with MYO6 at the TBCs at the Sertoli-spermatid interface. In mammalian cells, several MYO6 cargo adaptors have been identified mediating the function of this myosin in endocytosis, autophagy, and regulation of actin dynamics [14, 29]. Our analysis showed that two of these MYO6 interactors—TOM1/L2 and GIPC1—localized to the same TBC compartments as MYO6 (Figure 2F and G). Both adaptor proteins are also recruited to APPL1-positive endosomes in other cell types and tissues. TOM1/L2, similar to MYO6, was most pronounced in the area of TBC clustering where endocytosis occurs (Figure 2F.a–a”; *arrows* indicating TBCs), either as one bright spot or as several separate vesicles (Figure 2F.b–b”; *arrows* indicating TBCs). Next, we addressed the localization of GIPC1, which is known to co-localize with TOM1/L2 and MYO6 on APPL1-positive endosomes [30]. Indeed, also at spermatid/Sertoli cell interface, the regions positive for GIPC1 correlated with the localization of TOM1/L2 and MYO6 in the small concavity of spermatid heads (Figure 2G.a and b). Interestingly, GIPC1 localization was not restricted to the vesicles in the center of the TBC clusters, but similar to MYO6 was also present at the actin cuffs along the TBCs (Figure 2G.a’, a”, b’, and b”; *arrowheads* and *arrows*). Next, we assessed the expression levels of these three proteins, APPL1, TOM1/L2 and GIPC1, in the control and *sv/sv* testes by western blotting (Figure 3A). All three MYO6-binding partners are present at similar levels in control and *sv/sv* testis, indicating that their expression is not affected by the loss of MYO6. Furthermore, in co-immunoprecipitation experiments, affinity purified polyclonal antibodies to MYO6 were able to pull down MYO6 together with TOM1/L2 or GIPC1 from *sv/+* mouse testes (Figure 3B). No signal was observed in lysates from *sv/sv* testes used as a negative control. Together, these results indicate that in mouse testes, MYO6 is present in a complex with its binding partners TOM1/L2 and GIPC1, which recruit MYO6 to an APPL1-positive vesicular compartment associated with the TBCs.

### Ultrastructural localization of MYO6 and TOM1/L2 at the TBCs

**Figure 4:**
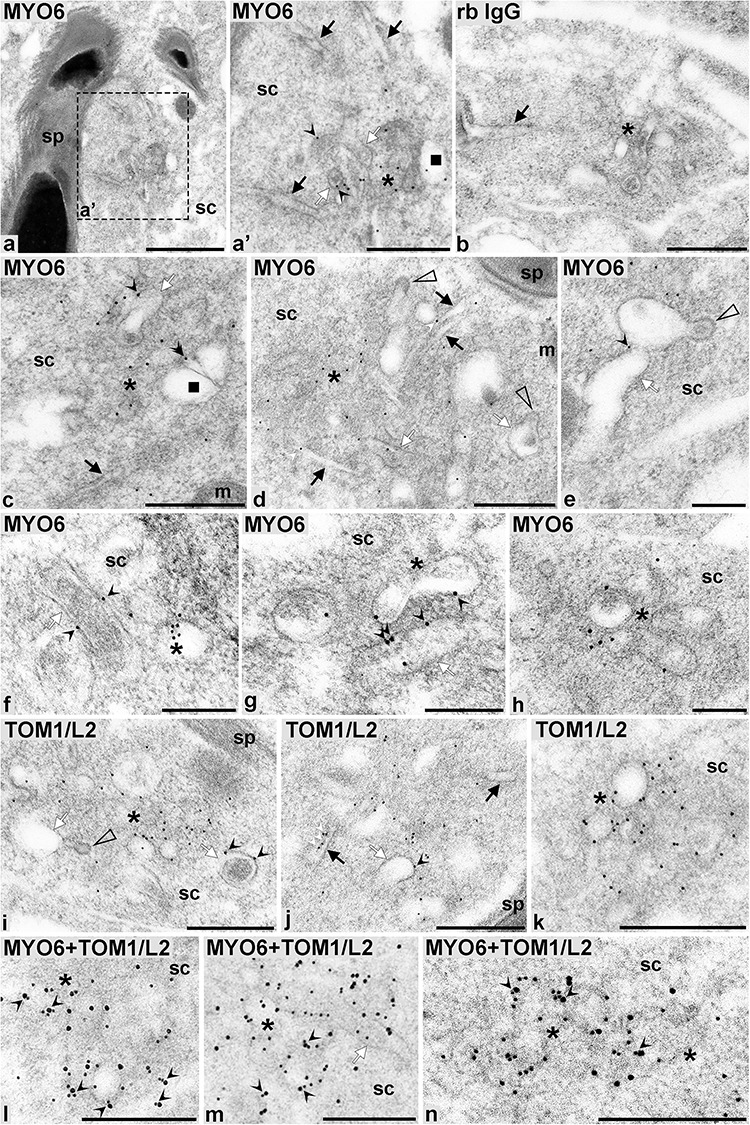
MYO6 and TOM1/L2 co-localize on vesicular structures and at the bulbar region of the TBCs. Detailed immunogold localization of MYO6 (a, a’, c–h), TOM1/L2 (i–k), double-localization of MYO6 and TOM1/L2 (l–n), and negative control (b) in *sv/+* spermatids. On, images *l-n* MYO6 was localized with 10 nm and TOM1/L2 with 15 nm gold particles. *Asterisks:* area enriched in endocytic structures, *black arrows:* proximal tubules of TBCs, *black squares:* larger endosomes, *white arrows:* bulbs of TBCs, *white arrowheads:* clathrin-coated pits of TBCs, *sc:* Sertoli cell, *sp:* spermatid. *Bars:* 1 μm (a), 500 nm (a’, b–d, i, j), 250 nm (e–h, k–n).

To determine the distribution of MYO6 at the TBCs at the ultrastructural level, we performed post-embedding immunogold labeling on *sv/+* testes sections using our polyclonal antibody to MYO6 followed by gold-conjugated protein A. Although the weaker fixation protocol required for immunocytochemistry is not optimal for preserving the ultrastructure of cellular organelles, our post-embedding method allowed the visualization of the TBCs structure at high resolution (Figure 4a–h). Using this technique, we were able to visualize the proximal tubules (*black arrows*) and the bulbular region (*white arrows*) of TBCs, as well as clathrin-coated pits (*empty arrowheads*), endocytosed bulbs/early endosomes (*asterisks*), and early sorting endosomes most probably corresponding to EEA1-positive compartment (*black squares*). On ultrathin sections of *sv/+* mouse testis that were stained with anti-MYO6 antibody, we observed gold particles mainly associated with vesicular structures in the center of the TBCs, thus confirming our results obtained by immunofluorescence (Figure 4a’, d, h; *asterisks*). Our analysis further revealed that to a lesser extent, MYO6 labeling is also present on the surface of the bulbs of the TBCs (*white arrows*) before their scission (Figure 4a’, c, e–g; *black arrowheads*) and on the long, proximal tubules of TBCs (Figure 4d; *white arrowheads*—gold particles, *black arrows*—tubules). Very few gold particles were found on structures most probably corresponding to EEA1-positive early sorting endosomes (Figure 4c; *double arrowhead*—gold particles, *black square*—early endosome), and no gold particles were present on clathrin-coated pits of TBCs (Figure 4d and e; *empty arrowheads*). No signal was observed at the TBC compartment in the sections labeled with normal rabbit IgG instead of the primary antibody (Figure 4b).

We next analyzed the localization of the MYO6-binding partner TOM1/L2 at the ultrastructural level to determine whether both proteins are present in the same cellular compartment/s. Using the immunogold labeling technique, we were able to confirm that TOM1/L2 localizes to the same TBC sub-compartments as MYO6 (Figure 4i–k). Consistent with our previous immunofluorescence results, we found that gold particles representing the localization of TOM1/L2 were mainly present in the area of the TBCs, where clusters of endocytic structures accumulate (Figure 4i and k; *asterisks*). Moreover, gold particles highlighting TOM1/L2 localization were associated with outer membranes of TBC bulbs (Figure 4i and j; *black arrowheads—*gold particles, *white arrows—*bulbs), and some gold particles were also found at the long, proximal tubules of TBCs (Figure 4j; *white arrowheads—*gold particles, *black arrow*—tubule). Similar to the result for MYO6, no TOM1/L2 was present at the clathrin-coated pits of TBCs (Figure 4i, *empty arrowhead*).

**Figure 5:**
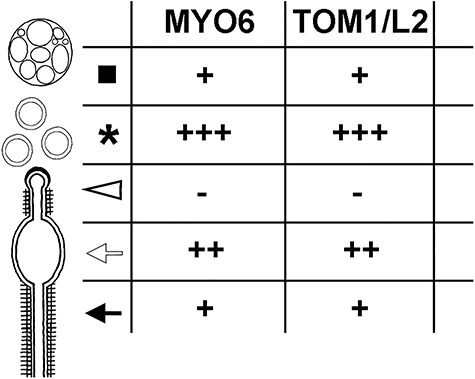
MYO6 and TOM1/L2 localize mainly to TBC bulbs prior and shortly after their internalization. The number of gold particles showing the localization of MYO6 or TOM1/L2 in different sub-compartments of TBCs on all acquired images scored as follows: + ≤ 10 gold particles, ++ ≤ 100 gold particles, +++ > 100 gold particles.

The use of gold-conjugated protein A, which binds only to a single site on the IgG molecule, enabled us to perform double-labeling experiments with two primary rabbit antibodies against MYO6 and TOM1/L2. For this experiment, we labeled the testis sections with the MYO6 and TOM1/L2 antibodies sequentially and detected the first antibody using two different protein A probes—the first conjugated with 10 nm gold particle for MYO6 and the other with 15 nm particle for TOM1/L2. As shown in Figure 4l–n, both proteins (*black arrowheads*) co-localize in the area of TBC clustering (*asterisks*), where early endosomes accumulate. Finally, we calculated the number of gold particles labeling MYO6 or TOM1/L2 to determine whether MYO6 and TOM1/L2 localize to the same sub-compartments of the TBCs (Figure 5). Our scoring shows that both proteins are preferentially associated with the bulbular region of the TBCs and the bulbs/early endocytic vesicles after their scission; this localization is similar to the one previously observed for Rab5 [6]. Moreover, both MYO6 and TOM1/L2 also associate to long proximal tubules and with early sorting endosomes corresponding to EEA1-positive compartment. Finally, both proteins are absent from clathrin-coated pits at the ends of the TBCs. Altogether, based on data obtained with immunogold electron microscopy, we conclude that MYO6 co-localizes with its binding partners mainly to the bulbs of the TBCs prior and shortly after their scission.

### MYO6 maintains the proper structure of the TBC compartment

**Figure 6:**
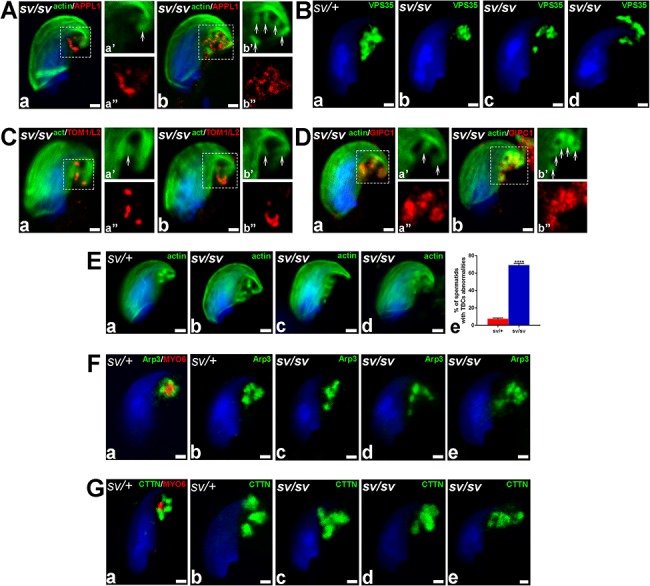
Lack of MYO6 in *sv/sv* spermatids leads to the dispersion of TBC endocytic compartment. (A) Confocal microscope images of stage VII *sv/sv* spermatids immunostained for APPL1 (*red*) and F-actin (*green*). (B) Confocal microscope images of stage VII *sv/+* and *sv/sv* spermatids immunostained for VPS35 (*green*). (C) Confocal microscope images of stage VII *sv/sv* spermatids immunostained for TOM1/L2 (*red*) and F-actin (*green*). (D) Confocal microscope images of stage VII *sv/sv* spermatids immunostained for GIPC1 (*red*) and F-actin (*green*). (E) Confocal microscope images of stage VII *sv/+* and *sv/sv* spermatids stained for F-actin (*green*) (a–d). The graph depicting the mean percentage of cells which displayed disturbed TBC-associated cytoskeleton (e). > 90 cells from *n* = 3 independent experiments were counted (3 litter pairs of *sv/+* and *sv/sv* males). Error bars indicate SEM, ^****^P ≤ 0.0001. (F) Confocal microscope images of stage VII *sv/+* and *sv/sv* spermatids immunostained for MYO6 (a, *red*) and Arp3 (a–e, *green*). (G) Confocal microscope images of stage VII *sv/+* and *sv/sv* maturing spermatids immunostained for MYO6 (a, *red*) and cortactin (a–e, *green*). *Arrows* highlight the position of TBCs and *dotted squares* indicate areas magnified in images marked with prime and double prime. *All bars* 1 μm.

To establish whether MYO6 has a role in maintenance of the structure/morphology of the TBCs in mouse testes, we analyzed functional organization of the TBCs in *sv/sv* spermatids from the MYO6 KO mouse testes lacking MYO6 expression (Figure 2A.a). We first explored whether the absence of MYO6 at TBCs (Figure 2B.b) has an impact on the correct localizations of its adaptor complexes by labeling *sv/sv* spermatids with antibodies against APPL1, TOM1/L2, and GIPC1 (Figure 6A, C, and D). Our immunofluorescence results show that APPL1-positive vesicles were mislocalized and dispersed in MYO6-deficient cells (Figure 6A.a and b; *arrows* in a’ and b’ show TBCs) and no longer concentrate at the site of TBC clustering compared to control mice (compare Figures 6A.a and b and 2D.a and b). In addition, we assessed the localization of VPS35, the marker protein for sorting endosomes, in the mutant cells to determine whether the spatial organization of the late endocytic compartment is also affected in MYO6-deficient spermatids. Interestingly, compared to the control cells, where VPS35 was concentrated at the concave side of spermatids head (Figure 6B.a), in *sv/sv* spermatids, the signal corresponding to VPS35 was less pronounced at this compartment (Figure 6B.b–d). Next, we determined the localization of TOM1/L2 and GIPC1 at the mutant spermatid-Sertoli cell interface (Figure 6C and D, *arrows* indicate TBCs). In *sv/sv* spermatids, TOM1/L2 is still present at the concave side of spermatid head; however, the vesicular localization is much more dispersed and less concentrated compared to *sv/+* spermatids containing wild-type levels of MYO6 (Figure 6C.a and b, compare with Figure 2F.a and b). Similarly, in the *sv/sv* spermatids, staining for GIPC1 was also more dispersed and less focused compared to the control cells (compare Figures 6D.a and b and 2G.a and b). The GIPC1 signal was markedly diffused (Figure 6D.a” and b”) and interestingly, much more strongly overlapped with actin cuffs of the TBCs in the MYO6 KO cells (Figure 6D.a’ and b’; *arrows*). Together, these results indicate that in MYO6-deficient cells, the APPL1-positive early endocytic vesicles no longer cluster at the ends of TBCs, indicating that TBC functional organization is impaired in MYO6 KO male mice, which may impact on the central role of this specialized structure in endocytosis of intercellular junctions.

We next examined the organization of the actin cytoskeleton, which is crucial to maintain the elaborate structure of TBCs, in the MYO6 mutant cells and visualized F-actin using fluorescently labeled phalloidin (Figure 6E). As shown in Figure 6E.a, in control cells, actin surrounding the TBCs projects towards one spot, where we observed the accumulation of APPL1 and MYO6-positive vesicles after cleavage. In *sv/sv* cells, however, labeling of the actin cytoskeleton revealed that the overall TBCs orientation is changed; they no longer clustered but pointed in different directions (Figure 6E.b) and sometimes were formed away from the concave surface of the spermatid head (Figure 6E.c). In some examples, we observed a very hazy phalloidin signal, suggesting the disruption of TBCs actin cuffs (Figure 6E.d). Quantification of cells exhibiting these phenotypes revealed that in *sv/sv* males, 69.25 ± 1.83% of spermatids/Sertoli cells had an abnormal TBC structure, whereas in control, this number was 7.65 ± 0.87% (possibly these results were partial due to the TBCs disruption during epithelium fragmentation and sample preparation) (Figure 6E.e). However, these results indicate a highly significant (*P* < 0.0001) almost tenfold increase in the number of cells lacking MYO6 with an abnormal TBC structure, visualized by F-actin staining.

Next, we analyzed in control cells the distribution of MYO6 and selected ABPs, Arp3, and cortactin, at the spermatid/Sertoli cell interface. These proteins that regulate actin dynamics are components of the mammalian TBCs [31, 32] and have been reported to co-localize with MYO6 in the actin cones during *Drosophila* spermiogenesis, where their distribution is disrupted in MYO6-deficient flies [19, 20]. We labeled control spermatids with antibodies specific for Arp3 and cortactin (CTTN on images) and found that both proteins were present at the TBCs specifically enriched in the actin cuffs along the proximal tubule (Figure 6F.a and G.a, respectively). We observed little overlap between MYO6 and Arp3 or cortactin, because MYO6 was concentrated in the early vesicular compartment at the center of the TBCs and to a lesser extent along the actin cuffs. We next analyzed the distribution of these ABPs in the *sv/sv* spermatids and observed that both Arp3 and also cortactin show in the absence of MYO6 much more diffuse staining patterns (Figure 6F.c–e and G.c–e) and less labeling along correctly organized TBC actin cuffs, as they can be found in control spermatids (Figure 6F.a and b and G.a and b). Altogether, these data show that MYO6 depletion impairs the development and regulation of TBC-associated cytoskeleton, which can be visualized by the staining for AFs forming the TBC-associated actin cuffs or labeling of the associated actin-regulatory proteins, Arp3 and cortactin.

### The loss of MYO6 impairs the spatial organization of nectin-3-containing endosomes at theTBCs

**Figure 7:**
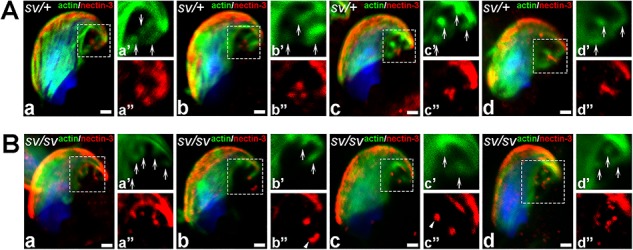
Lack of MYO6 in *sv/sv* spermatids disrupts the structural integrity of the nectin-3-positive endocytic compartment. Confocal microscopy images of stage VII *sv/+*(A) and *sv/sv* (B) spermatids representing dynamics of TBC-related endocytic compartment immunostained for nectin-3 (*red*) and F-actin (*green*). *Arrows* highlight the position of the TBCs and *dotted squares* indicate areas magnified in images marked with prime and double prime. *All bars* 1 μm.

Our results so far suggest that MYO6 is important for maintaining the integrity of the early endocytic compartment associated with the TBCs. To determine whether the loss of MYO6 may impair the endocytosis of intercellular junctions at TBCs, we determined the distribution of nectin-3, an adhesion junction protein in the spermatid membrane, which forms heterotypic cell-cell adhesions with nectin-2 present in the Sertoli cell membrane, in control and mutant spermatids (Figure 7, *arrows* indicate TBCs) [5, 6, 33]. As previously reported, nectin-3 was found in the apical ES that overlapped with F-actin bundles surrounding the spermatid heads (Figure 7.A and B; notice yellow/orange area indicating co-localization of nectin-3 and F-actin). In addition, nectin-3 appeared at/around the TBC compartment as dots/vesicles at the ends of TBCs (Figure 7A.a–a” and b–b”) or in small clusters (Figure 7A.c–c” and d–d”), which may represent adhesion junctions of the apical ES in early endosomes after internalization. In *sv/sv* spermatids, nectin-3 was also associated with vesicular structures; however, these were more dispersed and no longer concentrated at the site of TBCs clustering (Figure 7B). We did not observe any changes in the staining of nectin-3 at the apical ES. In summary, our immunofluorescence analysis indicates that the absence of MYO6 disturbs the steady state organization of endocytic machinery. Although our analysis of the fixed testes does not allow to quantify endocytosis of nectin-3, our data suggest that the observed disruptions in the spatial organization of the endocytic TBC may impact on the endocytosis of nectin-3-containing intercellular junctions in developing/maturing mouse spermatids.

## Discussion

Although the actin cytoskeleton and different ABPs have been shown to play important role/s at the late stage of mammalian spermiogenesis [1], molecular function of these cytoskeletal components and their temporal and spatial regulation are still poorly understood. Here we demonstrate that MYO6 is important to maintain the integrity of the actin-rich TBC compartment and the distribution of the endocytic machinery in Sertoli cells during the late phase of spermiogenesis in mice.

### MYO6 is essential for the spatial organization of endosomes at the TBC

MYO6 has previously been shown to localize to Rab5- and APPL1-positive early endosomes in the actin-rich cortex below the plasma membrane [26, 29, 34]. APPL1 is a multifunctional adaptor protein that interacts through its phosphotyrosine-binding domain with many different signaling receptors [35]. Interestingly, these early APPL1-positive vesicles are distinct from EEA1-positive early sorting endosomes. MYO6 is recruited to APPL1-endosomes through interactions with the adaptor proteins, GIPC1 and TOM1/L2, and the MYO6/GIPC1 complex enables the translocation of APPL1-endosomes through the actin cortex below the plasma membrane [26, 36]. The exact cellular functions of TOM1/L2 are less well understood; however, the MYO6-TOM1/L2 complex has been shown to facilitate the maturation of autophagosomes enabling their fusion with the lysosome [26, 30]. Overall, MYO6 plays a crucial role in tethering of early endosomes to cortical AFs enabling the maturation of nascent endosomes and the regulation of downstream signaling, which precedes the cargo processing in EEA1-positive early endosomes [34].

Based on our results, MYO6, GIPC1, and TOM1/L2 are not present in EEA1-positive sorting endosomes, which are marked by the presence of VPS35 [28], but are recruited to the bulbular region of the TBCs and associated to early endosomes. The nature of these endosomes is further characterized by the localization of Rab5 in rat testis, where this GTPase is present at the TBCs and on endosomes in close vicinity [6]. Since APPL1 is an effector of Rab5, both proteins are likely to be present in similar compartments. Therefore, the APPL1-positive TBC compartment containing MYO6, GIPC1, and TOM1/L2 in mouse testis may correspond to Rab5-positive compartments observed in rat testis. Given that we identified the same MYO6 adaptor proteins associated with the TBCs that were previously shown to bind to MYO6 in the endocytic pathway in other animal cells, MYO6 may also be involved in the early endosome distribution, cargo sorting, and endosome trafficking during the late phase of spermiogenesis in mouse. This hypothesis is also supported by our observation that the no-insert and small-insert MYO6 isoforms are expressed in mouse testis [21] and is in line with results showing that in other cell types/tissues the no-insert MYO6 localizes to early endosomes [30, 37, 38]. In summary, although the exact function of MYO6 in endocytosis at the TBCs is not known, the lack of MYO6 impacts the TBCs functional organization, which may affect downstream signaling essential for endocytic cargo processing and recycling. As a result, this may either affect the formation of new intercellular attachments formed in seminiferous epithelium or impair the final maturation and accurate sperm release to the lumen of seminiferous tubules.

### MYO6 might regulate actin dynamics that is crucial for spermatid maturation

Our data show that MYO6 is not only present at the APPL1-endosomes and the bulbular region of the TBCs but is also associated with AFs that cuff the proximal tubules of the TBCs. Interestingly, in MYO6-depleted testis, these actin-related structures are significantly disorganized, suggesting a role for this myosin in regulating the arrangement of AFs in the TBCs. Here, MYO6 could stabilize the actin network by recruiting or indirectly anchoring selected ABPs similar to its function in actin cones during *Drosophila* spermatid individualization [17]. During individualization, actin cones drive the removal of excess cytoplasm from maturing spermatids and membrane remodeling, and MYO6 concentrates at the front of these cones in the actin meshwork together with Arp3 and cortactin [18, 19]. The lack of MYO6 in developing *Drosophila* spermatids leads to the disturbed distribution of these ABPs and abnormal structure of actin cones, and as a result, to male infertility [17, 19]. Interestingly, not only in flies, but also in mice, depletion of MYO6 disrupts the localization of Arp3 and cortactin during spermiogenesis, suggesting that MYO6 may play a role in regulating actin organization/dynamics during spermatid development/maturation. Importantly, TBCs form in the area previously occupied by the apical ES [7]. Before the spermiation, the apical ES contains parallel actin bundles, which are reorganized into actin meshwork during the sperm release [39]. A similar F-actin remodeling process involving the Arp2/3 complex and its activator cortactin also takes place during *Drosophila* spermatid individualization [17–19]. Thus, we propose that the same or similar mechanisms involving the regulation of ABPs distribution by MYO6 could function at the late phase of spermiogenesis in mouse. The molecular mechanism that leads to disruption of Arp3 and cortactin localization after depletion of MYO6 is not clear, as neither of these regulatory ABPs bind directly to this myosin. MYO6, however, has been shown to interact via GIPC1 with LARG, a RhoGEF that induces actin polymerization and may modulate actin organization around cortical endosomes [29]. Furthermore, DOCK7, a GEF for Rac and CDC42 is linked indirectly to MYO6 through LRCH3 and the MYO6-LRCH3-DOCK7 complex promotes the displacement of septins that align on AFs [29, 40, 41]. Thus, MYO6 may regulate actin organization during spermiogenesis through recruitment of different RhoGEFs, thereby modulating the formation of distinct actin structures that require ABPs activity. Although both these RhoGEFs are expressed in mouse testis (data not shown), at present, their distributions and the other members of the septin family during spermiogenesis are not known.

Myosin VIIa, a plus-end-directed myosin motor, was previously showed to be linked to the apical ES in mammals, where it was postulated to be involved in spermatid and organelle transport and adhesion during spermiogenesis [11, 12]. However, myosin VIIa mutant mice revealed no obvious structural disruptions in the apical ES, suggesting that spermiogenesis progressed normal [42]. In contrast, work by Wen et al. [12] clearly showed that knock down of myosin VIIa in rat Sertoli cells induced disorganization of the actin cytoskeleton across the seminiferous tissue and abnormal spatio-temporal expression of selected ABPs just before and during spermiation. In addition, premature release of round/elongated spermatids and numerous defects in spermatozoa were noted in myosin VIIa-deficient cauda epididymis, suggesting serious problems in cell adhesion and spermatid transport during spermiogenesis in rat. All these findings suggest that multi-protein complexes involving different motor proteins that interact with the underlying actin cytoskeleton and various ABPs play important roles during spermiogenesis in mammals. We propose that MYO6 is one of the components of the protein complex at the TBCs involved in endocytosis at the spermatid/Sertoli interface during the late phase of spermiogenesis in mouse (summarized in graphical abstract).

## Conclusions

In summary, our data show that changes in the actin organization and distribution of the APPL1-positive endosomal compartment may lead to disruptions of TBCs in the testis of MYO6-deficient Snell’s waltzer mice. These defects may affect the sperm release and impair their fertilizing capacity, therefore causing the observed drop in fertility of the male mice. The phenotypes found in *sv/sv* spermatids suggest that in mammals, similar to invertebrates, MYO6 may have a structural role during spermiogenesis by regulating actin organization and tethering of membrane compartments to the surrounding actin cytoskeleton. It must be noted, however, that in contrast to *Drosophila* in which the lack of MYO6 expression leads to male infertility, in MYO6-deficient mice, the phenotype is less pronounced. Although MYO6 does not play an essential role in mammalian spermatogenesis, its function and mechanism of action during spermiogenesis are crucial to understand a molecular role of MYO6 in highly specialized tissues and cell types as found in the testes.
